# N6-methyladenosine demethylase FTO promotes growth and metastasis of gastric cancer via m^6^A modification of caveolin-1 and metabolic regulation of mitochondrial dynamics

**DOI:** 10.1038/s41419-022-04503-7

**Published:** 2022-01-21

**Authors:** You Zhou, Qi Wang, Haifeng Deng, Bin Xu, Yi Zhou, Jian Liu, Yingting Liu, Yufang Shi, Xiao Zheng, Jingting Jiang

**Affiliations:** 1 Tumor Biological Diagnosis and Treatment Center, The Third Affiliated Hospital of Soochow University, Changzhou, 213003 China; 2 Jiangsu Engineering Research Center for Tumor Immunotherapy, Changzhou, 213003 China; 3 Institute of Cell Therapy, Soochow University, Changzhou, 213003 China; 4 The First Affiliated Hospital of Soochow University, State Key Laboratory of Radiation Medicine and Protection, Institutes for Translational Medicine, Soochow University Medical College, Suzhou, 215123 China

**Keywords:** Cancer genomics, Gastrointestinal diseases

## Abstract

Gastric cancer (GC) is the fifth most common tumor and the third most deadly cancer worldwide. N6-methyladenosine (m^6^A) modification has been reported to play a regulatory role in human cancers. However, the exact role of m^6^A in GC remains largely unknown, and the dysregulation of m^6^A on mitochondrial metabolism has never been studied. In the present study, we demonstrated that FTO, a key demethylase for RNA m^6^A modification, was up-regulated in GC tissues, especially in tissues with liver metastasis. Functionally, FTO acted as a promoter for the proliferation and metastasis in GC. Moreover, FTO enhanced the degradation of caveolin-1 mRNA via its demethylation, which regulated the mitochondrial fission/fusion and metabolism. Collectively, our current findings provided some valuable insights into FTO-mediated m^6^A demethylation modification and could be used as a new strategy for more careful surveillance and aggressive therapeutic intervention.

## Introduction

Gastric cancer (GC) is the fifth most common tumor and the third most deadly cancer worldwide, with almost half of global GC cases diagnosed in East Asia [1]. Most GC patients are first diagnosed in the late stage of malignant hyperplasia and metastasis [2]. Unfortunately, the prognosis for patients with advanced GC remains poor [3]. Therefore, it is urgently necessary to identify new biomarkers and therapeutic targets for the diagnosis and treatment of GC.

Dysfunction of energy metabolism is thought to be a distinct feature of cancer [4]. Cancer cells support the occurrence and development of malignant tumors through metabolic recombination, especially mitochondrial metabolism [5]. Glycolysis has long been considered as the main metabolic process for energy production and anabolic growth of cancer cells [6]. Although such insights are instrumental in the development of powerful imaging tools that are still used in clinics, it is now clear that mitochondria play a key role in tumorigenesis [7]. In addition to its central bioenergy role, mitochondria indeed provide a cornerstone for tumor anabolism, control of redox and calcium homeostasis, participation in transcriptional regulation, and regulation of cell death [8, 9]. Mitochondria are therefore promising targets for the development of new anticancer drugs [10, 11]. Moreover, it is critical to understand the molecular mechanisms of mitochondrial metabolism in GC in order to develop future diagnostic and therapeutic strategies.

As the most common internal chemical modification of RNA in eukaryotes, N6-methyladenosine (m^6^A) modification is a reversible process mediated by m^6^A methyltransferase methyltransferase-like 3 (METTL3), methyltransferase-like 14 (METTL14), tumor 1 associated protein (WTAP), and elimination of fat mass and obesity-associated protein (FTO) or alkylation repair homologous protein 5 (ALKBH5) [12, 13]. In mammals, such modification affects different aspects of RNA metabolism, leading to mRNA stability and splicing, translation efficiency, nuclear output, selective polyadenylation, and microRNA processing [14]. Recent studies have revealed the effects of m^6^A modification on many biological processes, including fertility, immune regulation, metabolism, stem maintenance, and differentiation [12, 15]. Importantly, RNA m^6^A modification has been reported to play a regulatory role in human cancers [16–18]. For example, ALKBH5 maintains the tumorigenicity of glioblastoma stem cell-like cells [14]. FTO regulates chemoradiotherapy resistance of cervical squamous cell carcinoma [19, 20]. METTL3 controls myeloid differentiation in normal hematopoietic cells and leukemic cells [21]. A growing number of studies have confirmed the role of m^6^A in a variety of malignancies [13, 21]. However, the exact role of m^6^A in GC remains largely unknown, and the dysregulation of m^6^A on mitochondrial metabolism has never been studied.

In the present study, we demonstrated that FTO, a key demethylase for RNA m^6^A modification, played a key role in promoting proliferation and metastasis in GC. Collectively, our findings provided some valuable insights into FTO-mediated m^6^A demethylation modification. Moreover, we also explored the molecular mechanisms underlying the GC metastasis by identifying downstream target genes and signals.

## Materials and methods

### Cell lines and cell culture

All cells used in this study were obtained from the Chinese Cell Bank of the Chinese Academy of Sciences (Shanghai, China). Human GC cell lines AGS and SGC-7901 were maintained in Dulbecco’s Modified Eagle Medium (DMEM) supplemented with 10% fetal bovine serum (FBS) and 1% penicillin/streptomycin solution. Lentivirus was used to establish individual stable cell lines. Small interfering RNA (siRNA) duplexes targeting FTO (5′-GCAGTGTATCTGAGGAGCTCCATAA-3′, 100 nM), caveolin-1 (5′-TGTGACGAAATACTGGTTTTAC-3′, 100 nM) and corresponding negative control (NC) oligonucleotides were transfected into cells using Lipofectamine 3000 (Invitrogen) according to the manufacturer’s instructions.

### Cell proliferation assay

Cell proliferation assay was performed using CCK-8 kit (Dojindo) as previously reported. Briefly, cells were seeded into a 96-well plate (100 μl/well) at a density of 1 × 10^4^ cells/ml and cultured at 37 °C in an incubator containing 5% CO_2_. Each well was added with 10 μl CCK-8 solution, followed by incubation at 37 °C for 2 h. The spectrophotometric absorbance at a wavelength of 450 nm was determined for each sample. All the experiments were performed in triplicate.

For soft agar colony formation assays, cells were suspended in DMEM containing 0.35% low-melting agar (Invitrogen) and 10% FBS and seeded onto a coating of DMEM containing 0.8% low-melting agar and 10% FBS. Plates were incubated at 37 °C in a humidified atmosphere containing 5% CO_2_. Colonies were counted after 14 days of incubation. Each experiment was conducted in triplicate.

### Total RNA m^6^A quantification

The total level of m^6^A in the treated GC cells was then determined using the EpiQuik™ m^6^A RNA Methylation Quantitative Kit (Epigentek, USA). Briefly, 200 ng RNA was added to each well, followed by the addition of a mixture of capture antibodies and detection antibodies. After several weeks of incubation, the m^6^A content was quantified at a wavelength of 450 nm and calculated according to the standard curve.

### Dot blotting analysis

The mRNA samples were dissolved in three volumes of RNA incubation buffer at 65 °C for 5 min and loaded onto an Amersham Hybond-N + membrane (GE Healthcare, USA) mounted on a Bio-Dot device (Bio-Rad, USA). The membrane was blocked with 5% skim milk and then incubated with a specific m^6^A antibody (1:1000, RRID:AB_151230) overnight at 4 °C, followed by incubation with HRP-conjugated immunoglobulin G for 1 h, and imaging was performed using an imaging system (Bio-Rad, USA).

### Methylated RNA immunoprecipitation (Me-RIP)

Total RNA or poly (A) + mRNA was isolated by the above-mentioned methods. The purified mRNA and magnetic bead-antibody complexes were then added to the IP buffer and incubated at 4 °C overnight. RNA was eluted with eluent and then purified. The expressions of MGMT and ANPG at the RNA level were determined by real-time quantitative PCR (qRT-PCR).

### qRT-PCR

RNA extraction and qRT-PCR were performed as previously described. The relative expressions of the target genes at the mRNA level were calculated by the 2^−ΔΔCT^ method. GAPDH was used as a housekeeping gene. Primer sequences were as follows: METTL3, 5′-CAAGCTGCACTTCAGACGAA-3′ (Forward); 5′-GCTTGGCGTGTGGTCTTT-3′ (Reverse); METTL14, 5′-CTGGGGAGGGGTTGGACCTT-3′ (Forward); 5′-CCCCGTCTGTGCTACGCTTC-3′ (Reverse); RBM15, 5′-TCCCACCTTGTGAGTTCTCC-3′ (Forward); 5′-GTCAGCGCCAAGTTTTCTCT-3′ (Reverse); WTAP, 5′-CTTCCCAAGAAGGTTCGATTGA-3′ (Forward); 5′-TCAGACTCTCTTAGGCCAGTTAC-3′ (Reverse); VIRMA, 5′-AATCCTGTGGGAAGATCAGC-3′ (Forward); 5′-ACACGTAAGGCAGTGGTAAG-3′ (Reverse); FTO, 5′-CCAGAACCTGAGGAGAGAATGG-3′ (Forward); 5′-CGATGTCTGTGAGGTCAAACGG-3′ (Reverse); ALKBH, 5′-CCAGCTATGCTTCAGATCGCCT-3′ (Forward); 5′-GGTTCTCTTCCTTGTCCATCTCC-3′ (Reverse); caveolin-1, 5′-TGCTGTCTGCCCTCTTTGGC-3′ (Forward); 5′-GTGGGTCACAGACGGTGTGG-3′ (Reverse); m^6^A caveolin-1, 5′-CGTTCCCATCCGGGAACAGG-3′ (Forward); 5′-GCCAAAGAGGGCAGACAGCA-3′ (Reverse); GAPDH, 5′-TGACTTCAACAGCGACACCCA-3′ (Forward); 5′-CACCCTGTTGCTGTAGCCAAA-3′ (Reverse).

### Western blotting analysis

The cells were directly lysed in 1× sodium dodecyl sulfate-polyacrylamide gel electrophoresis (SDS-PAGE) loading buffer and subjected to SDS-PAGE. The blots were sequentially incubated with primary and HRP-conjugated secondary antibodies. The immunoreactive bands were then visualized using a Chemiluminescence Detection Kit (Servicebio, Wuhan, China) and detected with an imaging system (Bio-Rad, USA). Antibodies against FTO (1:2000, RRID:AB_280081) and caveolin-1 (1:1500, RRID: AB_32577) were obtained from Abcam. Antibodies against Pink1 (1:1500, Cat#6946), MFN2 (1:1000, Cat#11925), p-Parkin1 (1:1500, Cat#36728), Parkin1 (1:1000, Cat#2131), Cytochrome c (1:2000, Cat#12963) and β-tubulin (1:1500, Cat#2146) were obtained from Cell Signaling Technology. Antibody against p-MFN2 (1:3000, Cat#ABC963) was obtained from Merck Millipore. Antibody against GAPDH (1:5000, Cat# 60004) antibodies was purchased from Proteintech.

### RNA-seq and data analysis

Total RNA from AGS cells with or without FTO depletion was subjected to HiSeq RNA-Seq. Transcriptome reads from RNA-Seq experiments were mapped to the reference genome (hg19) using the Bowtie tool. The gene expression level was quantified by the RSEM software package. *p* < 0.05 was considered statistically significant. The differentially expressed genes were subsequently analyzed for the enrichment of biological pathways using the ClusterProfiler package. Gene interaction network analysis was performed using STRING website (https://string-db.org/) and Cytoscape software (3.6.0).

### Wound-healing assay

The cell migration ability was assessed by using a wound-healing assay to examine the regulatory role of FTO and caveolin-1 in the migration ability of GC cells as previously described.

### Transwell invasion assay

The transwell culture system was adopted to examine the invasive ability of FTO and caveolin-1 in GC cells as previously described.

### Mitochondrial fission assay

Mitochondrial fission was analyzed by staining mitochondria with 100 nM MitoTracker Green (Molecular Probes, Eugene, OR, USA). Cells were fixed in 4% paraformaldehyde (PFA) for 15 min. and permeabilized with 0.2% Triton X‐100. Mitochondria were imaged using a laser scanning confocal microscope (Zeiss LSM 800, Dublin, USA).

### Tumor xenografts

Male BALB/c nude mice (5 weeks old) were purchased from SLAC Animal Center (Shanghai, China) and then used for the xenograft tumor model. The animal-related protocols were approved by the Institutional Animal Care and Use Committee of the Third Affiliated Hospital of Soochow University. For the tumor growth analysis, AGS cells were subcutaneously injected into nude mice, and then the tumor volumes were monitored every 5 days. Tumor volumes were estimated based on the length and width and calculated using the following formula: tumor volume = (length × width^2^)/2. About 1 month later, the nude mice were sacrificed, and then tumors were excised, pictured, and weighed. For the tumor metastasis analysis, AGS cells were injected into nude mice by Tail Vein. About 1 month later, the nude mice were sacrificed, and then lung with metastasis lesions were excised, pictured, and counted.

### Immunohistochemistry (IHC) assays

Tumor slides from xenograft models and tissue array kit were collected and then embedded by paraffin after fixed in 4% PFA. IHC analyses were performed using specific anti-FTO (1:300, RRID:AB_280081) and anti-caveolin-1 (1:300, RRID: AB_32577) antibodies.

### Measurement of oxidative phosphorylation

Real-time integrated cellular oxygen consumption rate (OCR) was measured using the Seahorse XF24 Extracellular Flux Analyzer (Seahorse Bioscience, North Billerica, MA, USA) as previously described. Briefly, AGS cells were treated with 10 μg/ml curcumin for 12 h, and 10^4^ cells were plated into the customized Seahorse cell plates. After the probes were calibrated, the OCR was detected with sequential injection of the following compounds regulating mitochondrial respiration: oligomycin (ATP synthase inhibitor; 1 μM), FCCP (uncoupler; 1 μM), rotenone (complex I inhibitor; 1 μM), and antimycin A (complex III inhibitor; 1 μM).

### Statistical analysis

Data were expressed as the mean ± SEM from at least three independent experiments. A two-tailed *t*-test (unpaired) was used for two-group comparisons. For multiple comparisons, ANOVA followed by the post hoc Bonferroni test was taken with GraphPad Prism® version 9.0 software (GraphPad Software, Inc., La Jolla, CA, USA). *p* values < 0.05 were considered statistically significant. Sample sizes of all experiments were predetermined according to our experience. No sample was excluded from the analyses. Animals were not randomly assigned, but the sex, strain and age of the mice were the same, and the data analysis was single masked. Investigators were not blinded to the group allocation during the experiment and outcome assessment. The number of replicates and statistical method were indicated in each figure legend.

## Results

### FTO expression is up-regulated in GC tissues and its prognostic value in GC

**Fig. 1:**
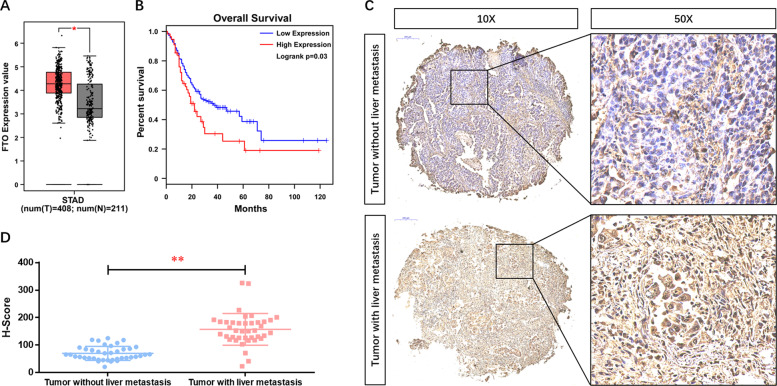
FTO expression is up-regulated in GC tissues and its prognostic value in GC. **A** The expression of FTO at the mRNA level and **B** the prognostic value in gastric cancer tissues were analyzed in the clinical database GEPIA (http://gepia.cancer-pku.cn/). **C** The expression of FTO at the protein level in the GC tissue microarray consisting of 90 cases was analyzed using IHC. **D** Quantitative analysis was performed upon the IHC data. *p < 0.05, **p < 0.01 compared with the indicated group.

To explore the expression profile of the major m^6^A eraser enzyme in GC, we first searched the clinical database GEPIA (http://gepia.cancer-pku.cn/) and found that the expression of FTO at the mRNA level in GC tissues was significantly higher compared with the normal tissues (Fig. 1A). Further analysis of survival data among metastatic individuals suggested that patients with high FTO expression had a shorter median survival time (Fig. 1B). We next examined the expression of FTO at the protein level in the GC tissue using a microarray consisting of 90 cases by IHC. Figure 1C shows that FTO was clearly located in the nucleus of GC cells. Besides, the positive expression of FTO was dominant in most GC liver metastasis tissues, which was further confirmed by quantitative analysis (Fig. 1D). Among them, 24 cases (26.67%) had weak staining, and 66 cases (73.33%) had strong staining. In addition, the expression of FTO was significantly increased with the increase of pN stage (*p* = 0.001), pT stage (*p* = 0.001), TNM stage (*p* < 0.001), and liver invasion (*p* = 0.008) (Table 1). Taken together, these data suggested that FTO was significantly involved in GC, especially in tumor liver metastasis, and it might be associated with metastatic progression of GC.

**Table 1:** Association between FTO expression and clinicopathological characteristics in gastric cancer.

Variables	FTO expression level		*p* Value
	Low	High	
Age			
≤60 years	4	36	0.135
>60 years	13	37	
Gender			
Male	12	54	1.000
Female	8	16	
T stage			
T1	0	5	*0.001*
T2	3	7	
T3	9	26	
T4	8	32	
N stage			
N0	14	17	*0.001*
N1	10	9	
N2	2	15	
N3	3	20	
M stage			
M0	24	45	*0.001*
M1	6	15	
TNM stage			
I/II	11	20	*<0.001*
III/IV	19	40	
Liver metastasis			
Negative	32	33	*0.008*
Positive	5	20	

Italic font signifies *p* < 0.05.

### Down-regulated m^6^A and elevated level of FTO are shown in GC liver metastasis tissues

**Fig. 2:**
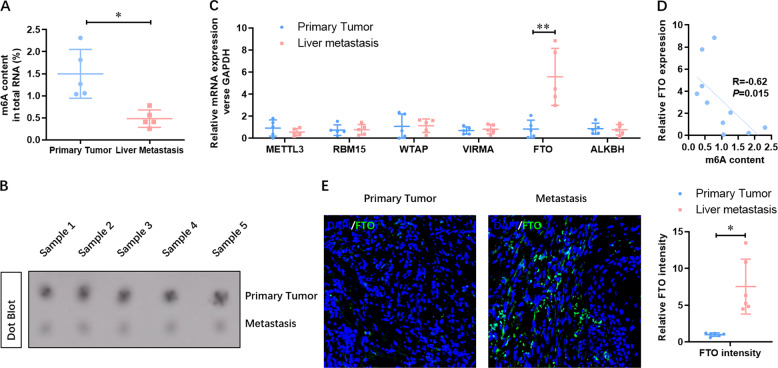
Down-regulated m^6^A and elevated level of FTO are shown in GC liver metastasis tissues. **A** The level of m^6^A methylated RNA in the tumor and liver metastasis tumor was analyzed by Colorimetric kit. **B** The level of m^6^A methylated RNA in the tumor and liver metastasis tumor was confirmed by m^6^A Dot blotting analysis. **C** The expression of FTO at the mRNA level was analyzed by qRT-PCR. **D** The level of m^6^A was negatively relative with FTO mRNA expression in the tumor and liver metastasis tumor. **E** The expression of FTO at the protein level was analyzed by IF. *N* = 5, *p < 0.05, **p < 0.01 compared with the indicated group.

To confirm the observation of the elevated level of FTO in GC liver metastasis tissues, we then constructed a liver metastasis model of GC in male C57BL/6N mice, and the GC liver metastasis tissues were collected. Compared with the generated subcutaneous tumor in male C57BL/6 NOD mice, the level of m^6^A methylated RNA was dramatically decreased (Fig. 2A), which was further confirmed by m^6^A Dot blotting analysis (Fig. 2B). Consistently, the expression of FTO at the mRNA level was significantly increased in GC liver metastasis tissues (Fig. 2C). Moreover, we found the level of m^6^A was negatively relative with FTO mRNA expression in the tumor and liver metastasis tumor (Fig. 2D). Meanwhile, the expression of FTO at the protein level in GC tissues was analyzed by IF, and we found a similar increase in GC liver metastasis tissues (Fig. 2E). These results suggested that down-regulated m^6^A and elevated FTO were found in GC liver metastasis tissues.

### Depletion of FTO impairs the proliferation and metastasis of GC cells in vitro and in vivo

**Fig. 3:**
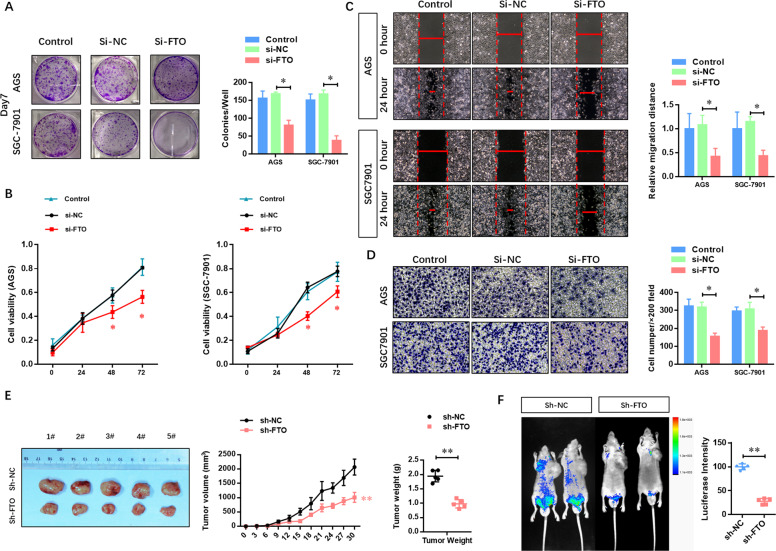
Depletion of FTO impairs the proliferation and metastasis of GC cells in vitro and in vivo. **A** The effect of FTO depletion on the colony formation ability of AGS and SGC-7901 cells was analyzed. **B** The effect of FTO depletion on the cellular viability of AGS and SGC-7901 cells was analyzed by CCK-8 assay. **C** The effect of FTO deletion on the migration ability of AGS and SGC-7901 cells was analyzed by wound-healing assay. **D** The effect of FTO depletion on the invasion ability of AGS and SGC-7901 cells was analyzed by transwell assay. **E** A subcutaneous tumor xenograft in Balb/c nude mice was established to analyze the effect of FTO on the proliferation of GC cells in vivo. **F** An orthotopic mouse model assay was performed to analyze the effect of FTO on metastasis. *N* = 3, *p < 0.05, **p < 0.01 compared with the indicated group.

To evaluate the biological function of FTO in GC cells, we then depleted FTO in AGS and SGC-7901 cells with specific siRNAs. Figure 3A shows that FTO depletion remarkably impaired the colony formation ability in AGS and SGC-7901 cells compared with the blank and NC groups. A similar suppressive effect was observed in the cellular viability (Fig. 3B), migration (Fig. 3C), and invasion (Fig. 3D) by CCK-8 assay, wound-healing assay, and transwell assay, respectively.

To determine the role of FTO in GC cells in vivo, AGS cells infected with shFTO and NC lentiviruses were used to establish a subcutaneous tumor xenograft in Balb/c nude mice. We observed the xenograft growth for 30 days and found that FTO depletion could significantly suppress the tumor growth in vivo (Fig. 3E). An orthotopic mouse model assay was also performed to further analyze the effect of FTO on metastasis. Luciferase-labeled AGS cells were injected beneath the serosa of the stomach of nude mice. Figure 3F shows that the number of metastatic nodes was dramatically decreased in FTO-depleted AGS cells. These results demonstrated that depletion of FTO impaired the proliferation and metastasis of GC cells in vitro and in vivo.

### Transcriptome-sequencing finds that FTO directly targets caveolin-1 mRNA and promotes its degradation

**Fig. 4:**
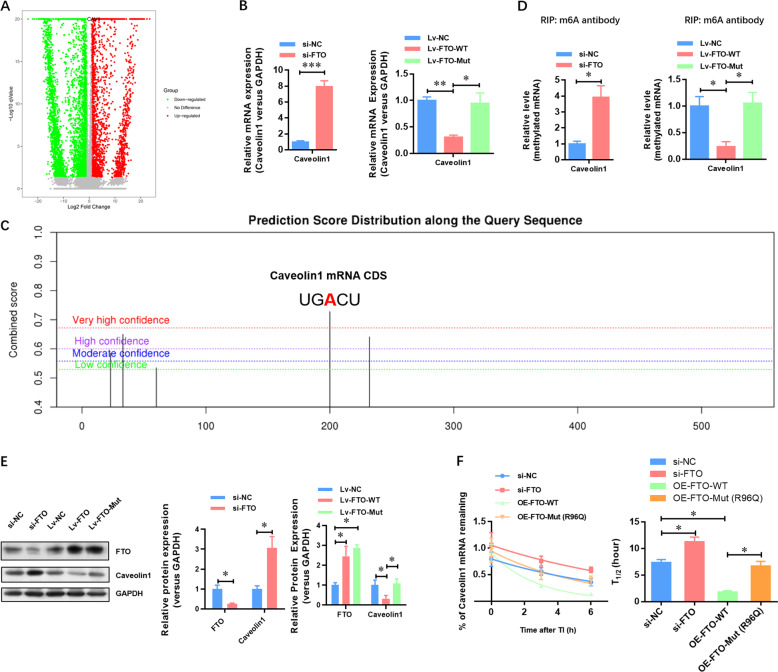
Transcriptome-sequencing finds that FTO directly targets caveolin-1 mRNA and promotes its degradation. **A** Caveolin-1 was up-regulated upon FTO depletion as indicated in volcano plot. **B** The elevation of caveolin-1 mRNA in FTO-depleted AGS cells was verified by qRT-PCR. **C** The m^6^A modification site predictor SRAMP was used to predict the m^6^A modification site in the CDS region of caveolin-1. **D** The elevation of m^6^A methylated caveolin-1 mRNA in FTO-depleted AGS cells was verified by Me-RIP-qPCR. **E** The elevation of caveolin-1 mRNA in FTO-depleted AGS cells was verified by Western blotting analysis. **F** Transcription inhibition assay showed that FTO depletion dramatically attenuated the degradation rate of caveolin-1 mRNA. *N* = 3, *p < 0.05, **p < 0.01 compared with the indicated group.

To investigate the functional implications of FTO and identify its potential targets in GC, we performed transcriptome-sequencing to compare the gene expression profile following FTO depletion in AGS cells. We found that 4852 genes were significantly down-regulated, while 3134 genes were significantly up-regulated (Fig. 4A and Supplementary Table 1). Among these potential top up-regulated genes, we focused on caveolin-1 due to its dramatic elevation (Fig. 4A). The elevation of caveolin-1 mRNA in FTO-depleted AGS cells was verified by quantitative reverse-transcription PCR (qRT-PCR) (Fig. 4B). Moreover, over-expression of wild-type FTO, not mutant FTO (R316A) lacking demethylation activity, decreased the expression of caveolin-1 at the mRNA level (Fig. 4B). Additionally, we found a very high confidence m^6^A modification site at the 200 bp of caveolin-1 CDS region using m^6^A modification site predictor SRAMP (Fig. 4C). Similar changes were observed in the methylated caveolin-1 mRNA by Me-RIP-qPCR (Fig. 4D). The changes in protein levels were also confirmed by Western blotting analysis (Fig. 4E). Transcription inhibition assay further showed that FTO depletion dramatically attenuated the degradation rate of caveolin-1 mRNA (Fig. 4F). In contrast, over-expression of wild-type FTO, not mutant FTO (R316A) lacking demethylation activity, enhanced the degradation rate of caveolin-1 mRNA (Fig. 4F). These data collectively suggested that FTO directly targeted caveolin-1 mRNA and promoted its degradation.

### FTO depletion impairs the proliferation, migration, and invasion of GC cells via inhibiting caveolin-1

**Fig. 5:**
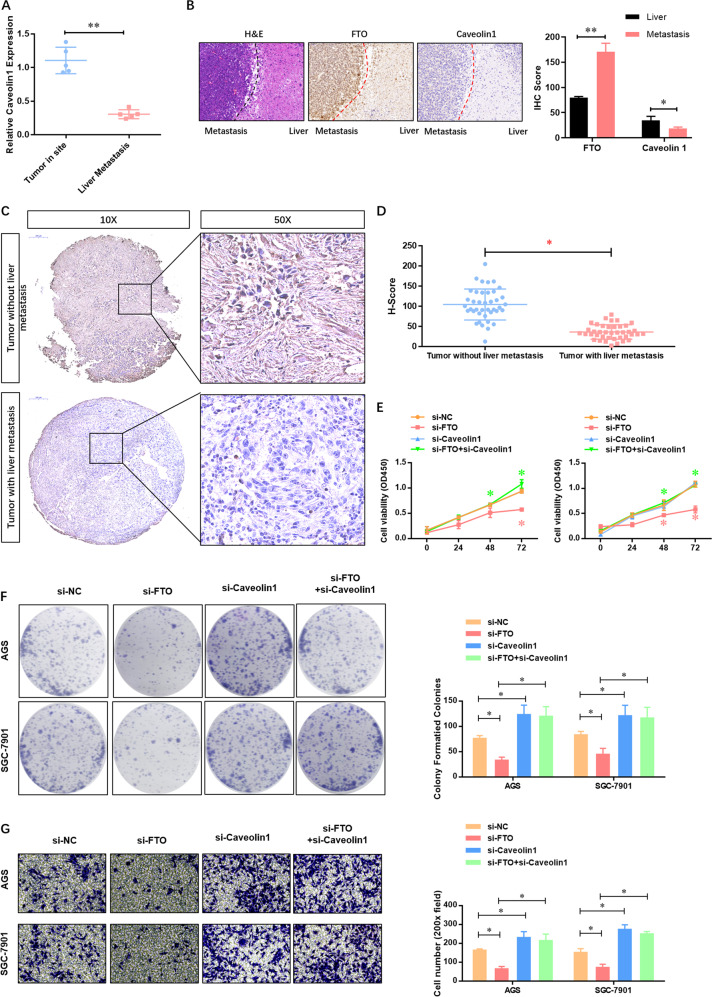
FTO depletion impairs the proliferation, migration, and invasion of GC cells via inhibiting caveolin-1. **A** The expression of caveolin-1 at the mRNA level in the tumor and liver metastasis tumor was analyzed by qRT-PCR. **B** The expression of caveolin-1 at the protein level in the tumor and liver metastasis tumor was analyzed by IHC. **C** The expression of caveolin-1 at the protein level in the GC tissue microarray consisting of 90 cases was analyzed using IHC, and **D** quantitative analysis was performed using the IHC data. **E** The effect of caveolin-1 depletion on the cellular viability of AGS and SGC-7901 cells with or without FTO depletion was analyzed by CCK-8 assay. **F** The effect of caveolin-1 depletion on the proliferation of AGS and SGC-7901 cells with or without FTO depletion was analyzed by colony formation assay. **G** The effect of caveolin-1 depletion on the invasion of AGS and SGC-7901 cells with or without FTO depletion was analyzed by transwell assay. *N* = 3, *p < 0.05, **p < 0.01 compared with the indicated group.

To further understand the role of caveolin-1 in the FTO-regulated biological functions of GC cells, we then analyzed the expression of FTO in liver metastasis. The result showed a low-expression level of caveolin-1 in GC liver metastasis tissues from mouse models by qRT-PCR (Fig. 5A) and IHC (Fig. 5B). We then confirmed these findings using the GC tissue microarray consisting of 90 cases by IHC. Figure 5C reveals that caveolin-1 was clearly located in the cytoplasm of GC cells. Besides, the positive expression of caveolin-1 was dominant in most GC tissues with no liver metastasis, which was further confirmed by quantitative analysis (Fig. 5D). Among them, 58 cases (64.44%) had weak staining, and 32 cases (35.55%) had strong staining. Besides, the expression of caveolin-1 was significantly decreased with the increase of pN stage (*p* = 0.004), pT stage (*p* < 0.001), TNM stage (*p* < 0.001), and invasion ability (*p* = 0.012) (Table 2). Notably, we found that caveolin-1 depletion did not affect the cellular viability (Fig. 5E), but elevated the colony formation (Fig. 5F) and invasion ability (Fig. 5G) in AGS and SGC-7901 cells. Moreover, caveolin-1 depletion significantly attenuated the FTO depletion-inhibited cellular viability (Fig. 5E), colony formation (Fig. 5F), and invasion ability (Fig. 5G) in AGS and SGC-7901 cells. Taken together, these data suggested that FTO impaired the proliferation, migration, and invasion of GC cells via inhibiting caveolin-1.

**Table 2:** Association between caveolin-1 expression and clinicopathological characteristics in gastric cancer.

Variables	Caveolin-1 expression level		*p* Value
	Low	High	
Age			
≤60 years	23	17	0.135
>60 years	26	24	
Gender			
Male	37	30	0.856
Female	13	10	
T stage			
T1	3	2	*<0.001*
T2	6	4	
T3	31	8	
T4	23	13	
N stage			
N0	16	9	*0.004*
N1	15	2	
N2	5	14	
N3	16	13	
M stage			
M0	35	34	*<0.001*
M1	11	10	
TNM stage			
I/II	26	9	*<0.001*
III/IV	37	18	
Liver metastasis			
Negative	35	20	*0.012*
Positive	25	10	

Italic font signifies *p* < 0.05.

### FTO depletion impairs the mitochondrial metabolism of GC cells via caveolin-1

**Fig. 6:**
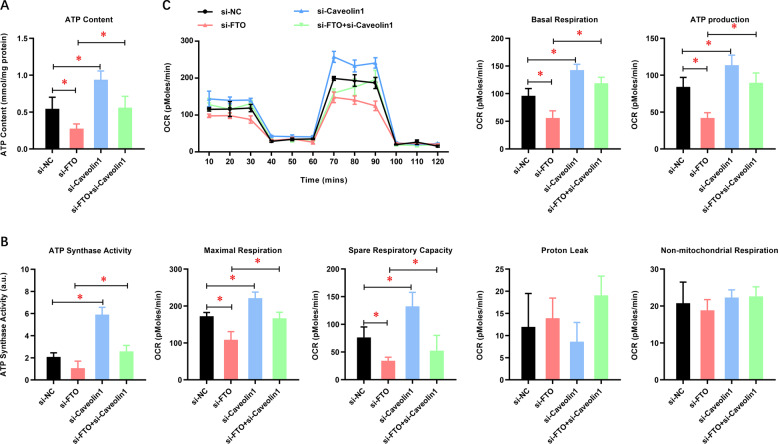
FTO depletion impairs the mitochondrial metabolism of GC cells via caveolin-1. **A** The effect of caveolin-1 depletion on the ATP content of AGS and SGC-7901 cells with or without FTO depletion was analyzed. **B** The effect of caveolin-1 depletion on the ATP synthase activity of AGS and SGC-7901 cells with or without FTO depletion was analyzed. **C** The effects of FTO and caveolin-1 on mitochondrial respiration (OXPHOS) were tested using the Seahorse XF24 Extracellular Flux Analyzer. *N* = 3, *p < 0.05, **p < 0.01 compared with the indicated group.

Mitochondria play an important role in the cellular biochemistry of most eukaryotic cells, which produce nearly 95% of cellular ATP through oxidative phosphorylation of mitochondria, thereby controlling cell death or survival. Considering the critical role of caveolin-1 in the regulation of mitochondrial metabolism, we then tested whether FTO promoted the biological functions of GC cells via caveolin-1-dependent regulation of mitochondrial metabolism. To test this hypothesis, we determined the effects of FTO and caveolin-1 on mitochondrial respiration (OXPHOS) using the Seahorse XF24 Extracellular Flux Analyzer. FTO depletion dramatically reduced the ATP content (Fig. 6A), ATP synthase activity (Fig. 6B), and cellular OCR (Fig. 6C), indicating reduced OXPHOS in AGS and SGC-7901 GC cells. Furthermore, we assessed specific mitochondrial functions, in particular basal respiration, maximal respiration, ATP production, spare respiratory capacity, proton leak, and non-mitochondrial respiration. FTO depletion dramatically impaired the OCR value of basal respiration, maximal respiration, ATP production, and spare respiratory capacity in AGS and SGC-7901 cells. In contrast, caveolin-1 depletion significantly enhanced the ATP content (Fig. 6A), ATP synthase activity (Fig. 6B), and cellular OCR (Fig. 6C). Furthermore, caveolin-1 depletion attenuated the inhibitory effect of FTO on the ATP content, ATP synthase activity, and cellular OCR. These data indicated that FTO depletion suppressed mitochondrial respiration via elevating caveolin-1, which might result in reduced ATP supplement and thereby restrict cancer cell growth.

### FTO depletion induces mitochondrial fission in GC cells via caveolin-1

**Fig. 7:**
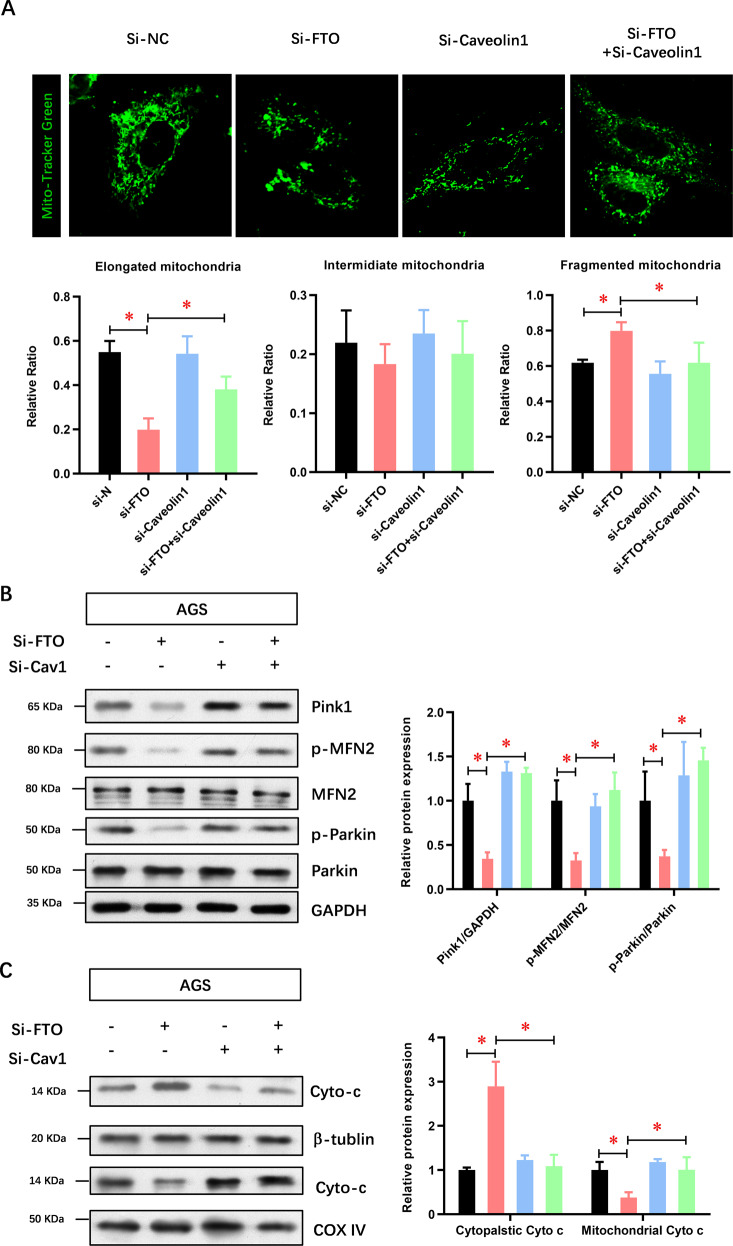
FTO depletion induces mitochondrial fission in GC cells via caveolin-1. **A** MTG staining and confocal microscopy were used to observe the mitochondrial fragmentation in AGS cells. **B** The regulatory molecules of mitochondrial fission and fusion, including Pink1, Parkin1, and MFN2, were analyzed. **C** The regulatory molecule of mitochondrial pathway apoptosis, indicated by cytochrome c release from mitochondria, was analyzed. *N* = 3, *p < 0.05, **p < 0.01 compared with the indicated group.

When mitochondria are destroyed by stresses, such as reactive oxygen species, the balance of mitochondrial dynamics is destroyed, leading to mitochondrial fission. Here, we determined whether FTO depletion altered the mitochondrial dynamics of cancer cells. MitoTracker Green staining and confocal microscopy were used to observe the cell morphology. Compared with untreated control cells, the proportion of mitochondrial fragments in FTO-depleted GC cells was increased, while the proportion of elongated mitochondria was decreased (Fig. 7A). Caveolin-1 did not change the morphology of mitochondria, whereas it reversed the FTO depletion-increased fragmented mitochondria and decreased elongated mitochondria (Fig. 7A). Mitochondrial fission and fusion are regulated by mitochondrial motion-related GTPases, such as Pink1, Parkin1, and MFN2, which thus induce the mitochondrial pathway of apoptosis via cytochrome c release from mitochondria. Figure 7B shows that FTO depletion significantly decreased the expressions of Pink1, phosphorylated Parkin1 and phosphorylated MFN2 at the protein level in AGS cells, which was similarly reversed by caveolin-1 depletion. Moreover, FTO depletion enhanced the release of mitochondrial cytochrome c into the cytoplasm, which was also reversed by caveolin-1 depletion (Fig. 7C). Accordingly, mitochondrial fission inhibitor Mdivi-1 significantly suppressed the cell proliferation of FTO overexpressed AGS and SGC-7901 cells (Supplementary Fig. 1). The above-mentioned results showed that FTO depletion induced mitochondrial fission in GC cells via caveolin-1.

### FTO depletion impairs the tumor growth via caveolin-1 in the xenograft model

**Fig. 8:**
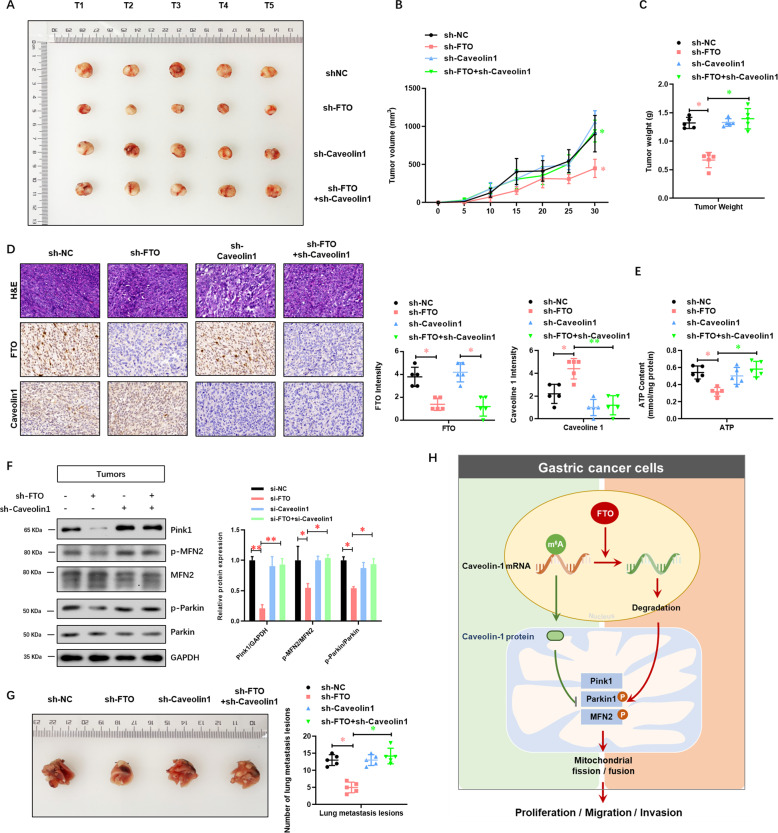
FTO depletion impairs the tumor growth via caveolin-1 in a xenograft model. **A** To further confirm the effect of FTO/caveolin-1 signaling on tumor growth in vivo, a subcutaneous tumor xenograft in Balb/c nude mice was established. **B** The curve of tumor growth and **C** tumor weight was analyzed. **D** The expressions of FTO and caveolin-1 at the protein level in these generated tumors were confirmed by IHC. **E** The ATP content in these generated tumors was analyzed. **F** The regulatory molecules of mitochondrial fission and fusion, including Pink1, Parkin1, and MFN2 in these generated tumors, were analyzed. **G** To further confirm the effect of FTO/caveolin-1 signaling on tumor metastasis in vivo, a Tail Vein injection tumor xenograft in Balb/c nude mice was established. The lung metastasis lesions from four groups of AGS cells were analyzed. **H** A working model demonstrates that the key demethylase of m^6^A FTO promotes the proliferation and metastasis of gastric cancer via the degradation of caveolin-1 mRNA and regulating the mitochondrial fission/fusion and metabolism. *N* = 5, *p < 0.05, **p < 0.01 compared with the indicated group.

To further confirm the effect of FTO/caveolin-1 signaling on tumor growth in vivo, a subcutaneous tumor xenograft in Balb/c nude mice was established. FTO depletion consistently inhibited the tumor growth (Fig. 8A, B) and decreased the tumor weight (Fig. 8C). Caveolin-1 depletion did not affect the tumor growth of AGS cells, whereas it completely reversed the FTO-inhibited tumor growth (Fig. 8A, B) and reduced the tumor weight (Fig. 8C). Furthermore, the expressions of FTO and caveolin-1 at the protein level in these generated tumors were confirmed by IHC (Fig. 8D). FTO depletion dramatically reduced the ATP content in the generated tumors, which was reversed by caveolin-1 depletion (Fig. 8E). Consistently, FTO depletion significantly decreased the expressions of Pink1, phosphorylated Parkin1 and phosphorylated MFN2 at the protein level in the generated tumors, which was similarly reversed by caveolin-1 depletion (Fig. 8F). To further confirm the effect of FTO/caveolin-1 signaling on tumor metastasis in vivo, a Tail Vein injection tumor metastasis xenograft in Balb/c nude mice was established. As shown in Fig. 8G, FTO depletion significantly decreased the number of lung metastasis lesion, which was reversed by caveolin-1 depletion. These results collectively suggested that FTO depletion impaired the tumor growth and metastasis via caveolin-1 in the xenograft model.

## Discussion

Methylation plays an important role in a variety of biological and pathological processes, including tumorigenesis [20, 22, 23]. Methylation is thought to regulate the unwinding and transcription of DNA, and with the increasing understanding of epigenetic control, the comprehension of nucleic acid methylation has been expanded from DNA to RNA. As the most common epigenetic modification on eukaryotic RNA, m^6^A is specified by methylation of adenosine and controlled by author, reader, and eraser [24, 25]. The degree of m^6^A modification is weakened by the writer enhancement or eraser, while the reader can recognize and play the role in m^6^A modification, although it does not directly affect the level of m^6^A [26]. RNA m^6^A modification has been reported to influence carcinogenesis [27–29], while its role in the regulation of GC carcinogenesis and progression remains largely undetermined.

FTO was originally thought to be a regulator of body weight and obesity because FTO deficiency leads to growth retardation, while FTO overactivation increases food intake and contributes to obesity [30]. After FTO is identified as the first RNA demethylation enzyme that can remove m^6^A from RNA through an a-ketoglutarate, it has been found to have new functions to control different aspects of biological processes, such as dopaminergic signaling and fat formation [31]. More importantly, there is growing evidence that FTO dysfunction can promote the development of cancers, such as acute myeloid leukemia (AML) [32], melanoma [33], breast cancer [34], and lung cancer [35]. FTO is considered as a prognostic factor of lung squamous cell carcinoma, promoting cell proliferation and invasion, but inhibiting cell apoptosis by regulating the expression of MZF1 [35]. It has been recently reported that FTO plays a critical oncogenic role in hematopoietic cell transformation and AML as an m^6^A demethylase [32]. Moreover, FTO plays critical roles in self-renewal and immune evasion of cancer stem cells, and small-molecule FTO inhibitors can exhibit strong anti-tumor effects in multiple types of cancer [36]. In the present study, we identified that the depletion of a critical m^6^A eraser FTO promoted the proliferation and metastasis in GC cells.

Caveolin-1 is a complete membrane protein that is expressed in large amounts in adipocytes, endothelial cells, lung cells, fibroblasts, and muscle cells [37]. Caveolin-1 plays an important role in cancer genesis because it is overexpressed or mutated in many solid human tumors [38–40]. However, the role of epithelial caveolin-1 in tumorigenesis remains controversial. Notably, caveolin-1 has been shown to act as an anti-apoptotic and pro-apoptotic protein, both as a tumor promoter and a tumor suppressor [41]. Besides, caveolin-1 can stimulate metastasis and serve as a prognostic marker [42]. Transcriptional analysis shows that many oncogenes down-regulate the expression of caveolin-1 [43]. Besides, the gene encoding caveolin-1 is located at a putative tumor suppressor gene locus. Finally, it has been hypothesized that caveolin-1 is a tumor suppressor [44]. However, loss of caveolin-1 expression has also been found to be associated with increased independent growth of the anchor, reflecting its metastatic potential. However, for some tumors, the up-regulation of caveolin-1 can affect the survival and growth of cancer cells and facilitate tumor progression. Herein, we found that caveolin-1 was critical for the FTO depletion-impaired cell proliferation and metastasis. FTO directly targeted the caveolin-1 mRNA and promoted its degradation.

In terms of mechanism, caveolin-1 is associated with the mitochondrial number and bioenergy function in various cell types [45]. For example, mitochondria of hypercholesterolemic rabbit hepatocytes are found to have a high caveolin-1 expression [46]. Compared with the control group, intravenous injection of antenna-caveolin-1 (AP-caveolin-1) peptide in mice increases the transmembrane transposition of caveolin-1, which results in a mitochondrial matrix with deeper electron density and a decrease in superoxide dismutase and catalase activities, suggesting that caveolin-1 is necessary for maintaining mitochondrial structure and function [41, 47, 48]. AP-caveolin-1 treatment also restores respiratory chain subunit protein (complex I-V), preserves mitochondrial function, and inhibits apoptotic cell death [49]. Colon cancer cells with over-expression of caveolin-1 (HCT116) also have abundant mitochondrial localization, and in low-expression cells (HT29), over-expression of caveolin-1 leads to its enrichment in mitochondria and reduced apoptosis [50]. These findings provide clues that caveolin-1 enhances mitochondrial function to support tumor progression, the details of which remain unclear. On the other hand, high expression of caveolin-1 in mitochondria inhibits the proliferation of H-Ras-driven tumor cells [51]. Specifically, tumor transformation triggered by tumorigenic H-RAS12V in NIH3T3 cells inhibits intracellular basal calcium (Ca^2+^) levels, Ca^2+^ influx, and caveolin-1 expression [51, 52]. Besides, reintroduction of caveolin-1 can enhance mitochondrial Ca^2+^ uptake, inhibit cell growth and colony formation, and induce cell apoptosis, suggesting that a high caveolin-1 level inhibits mitochondrial function, thereby mediating the inhibitory activity of H-Ras-driven tumors [53]. In our present study, we found that FTO depletion markedly induced mitochondrial fission and inhibited mitochondrial metabolism. However, these effects were dominantly reversed by caveolin-1 inhibition, suggesting that the effect of FTO depletion on the mitochondria relied on the up-regulation of caveolin-1.

In conclusion, this study demonstrated that the key demethylase of m^6^A FTO promoted the proliferation and metastasis of GC via regulating the mitochondrial fission/fusion and metabolism. In terms of mechanism, FTO improved the degradation of caveolin-1 mRNA via its demethylation (Fig. 8H). Collectively, our findings provided valuable insights into FTO-mediated m^6^A demethylation modification, which could be used as a new strategy for more careful surveillance and aggressive therapeutic intervention.

## Supplementary information

Supplementary Figure 1

Supplementary Table 1

Agreement from co-authors

Author Contribution Statement

Reproducibility Checklist form

Supplementary figure legend

## Data Availability

RNA-seq data supporting the results of this study has been deposited in the NCBI GEO database under accession number GSE178697.
