# Supplementary figures and images for: N6-methyladenosine demethylase FTO promotes growth and metastasis of gastric cancer via m6A modification of caveolin-1 and metabolic regulation of mitochondrial dynamics
Source: Cell Death Dis. 2022 Jan 21;13(1):72. doi: 10.1038/s41419-022-04503-7 (PMC8782929; doi:10.1038/s41419-022-04503-7)

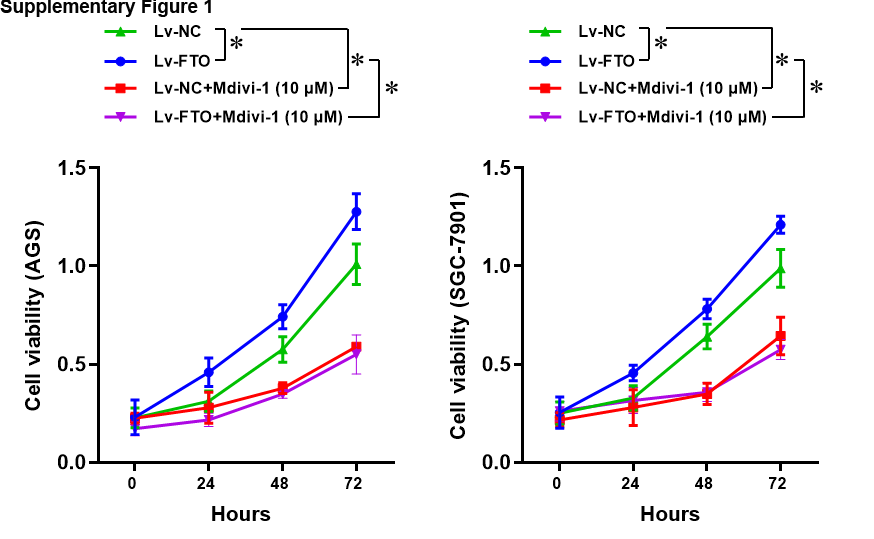

Supplement: Supplementary file 1 — Supplementary Figure 1 [file 41419_2022_4503_MOESM1_ESM.tif]
