# Supplementary material for: N6-methyladenosine demethylase FTO promotes growth and metastasis of gastric cancer via m6A modification of caveolin-1 and metabolic regulation of mitochondrial dynamics
Source: Cell Death Dis. 2022 Jan 21;13(1):72. doi: 10.1038/s41419-022-04503-7 (PMC8782929; doi:10.1038/s41419-022-04503-7)

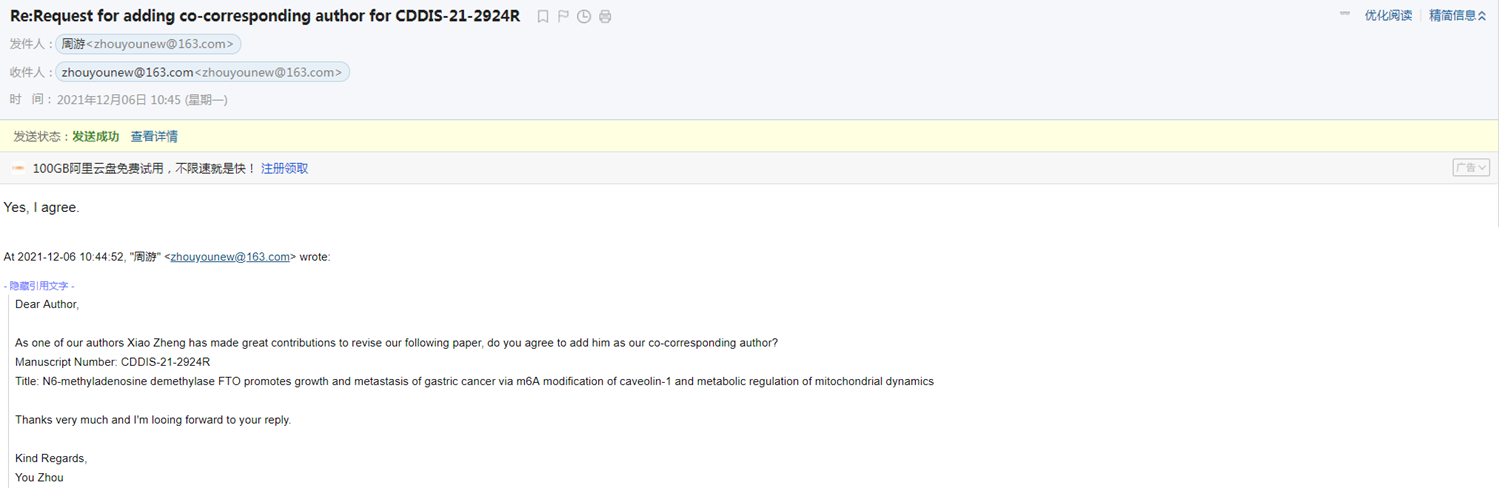
1. Agreement from co-author You Zhou:

2. Agreement from co-author Qi Wang:


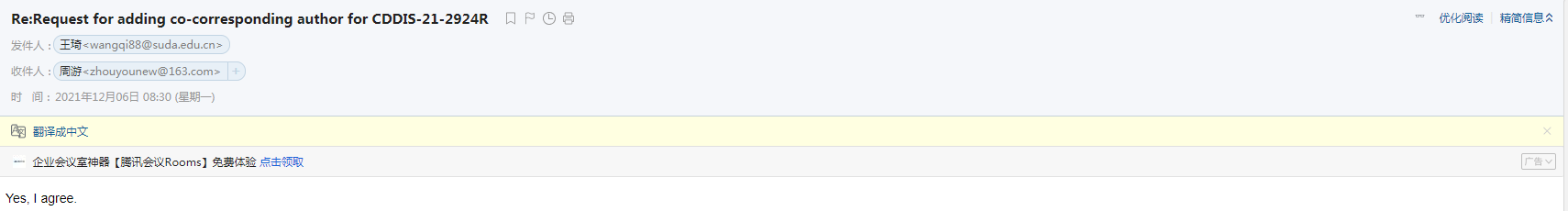

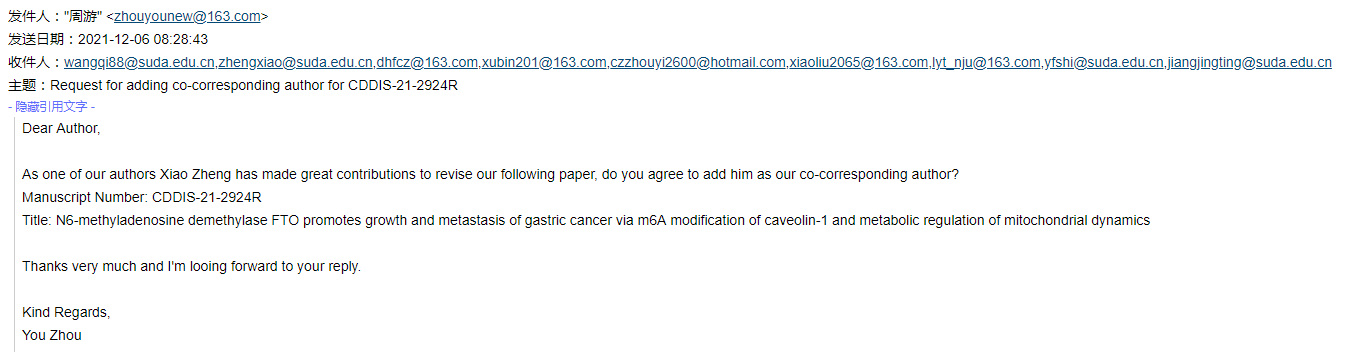


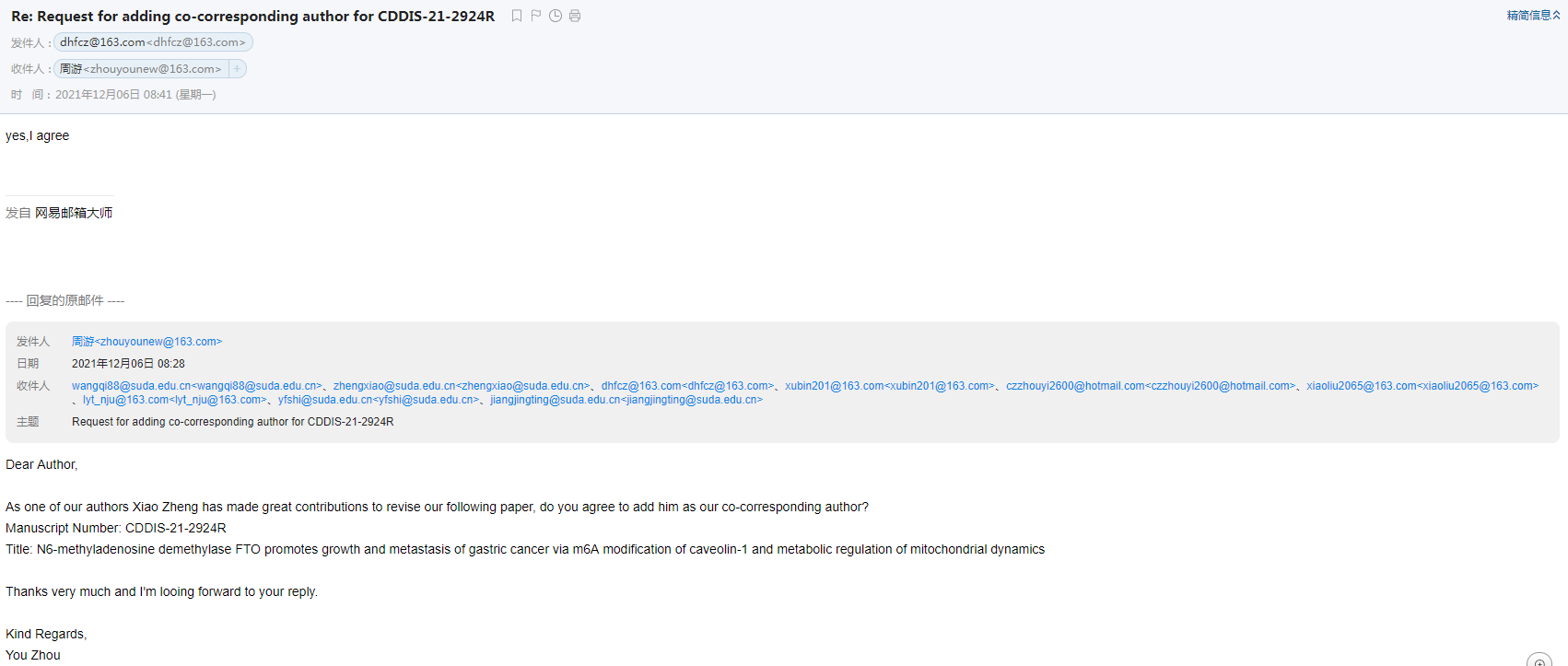
3. Agreement from co-author Haifeng Deng:

4. Agreement from co-author Xiao Zheng:


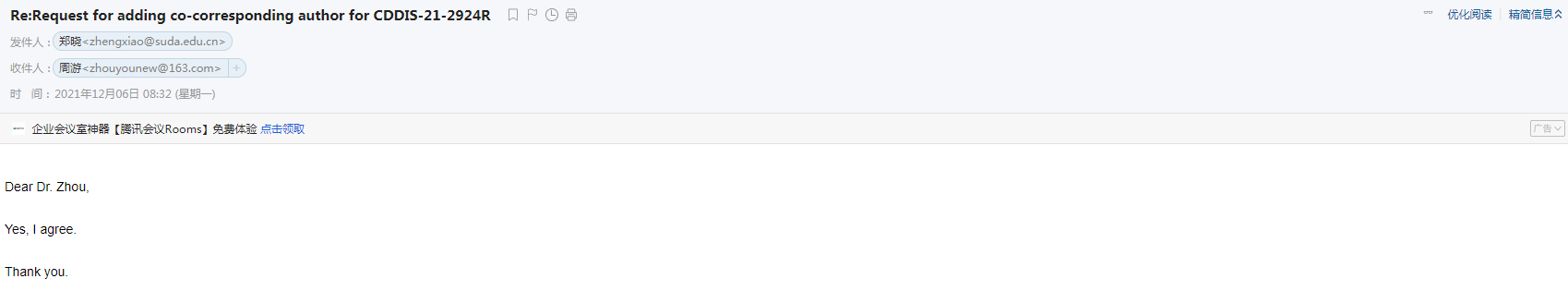

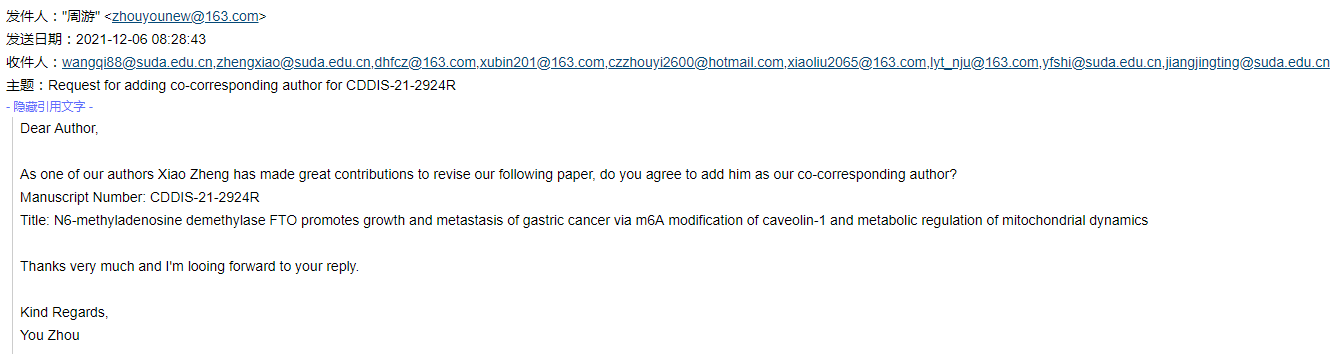


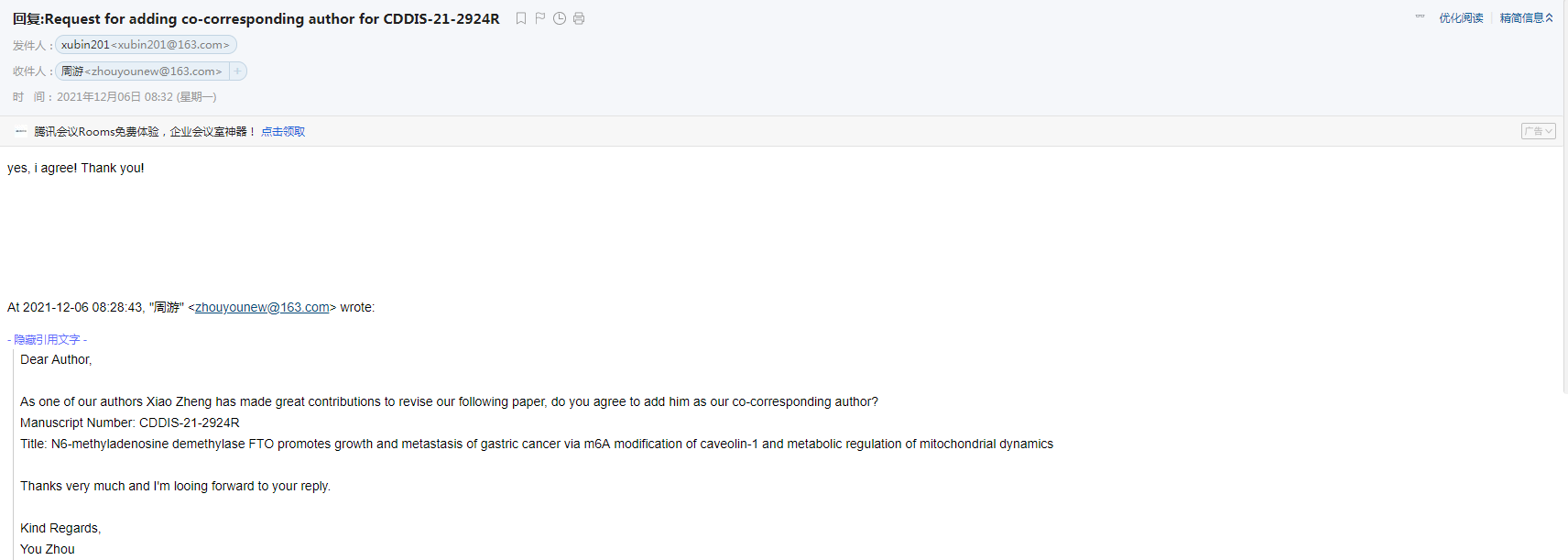
5. Agreement from co-author Bin Xu:

6. Agreement from co-author Yi Zhou:


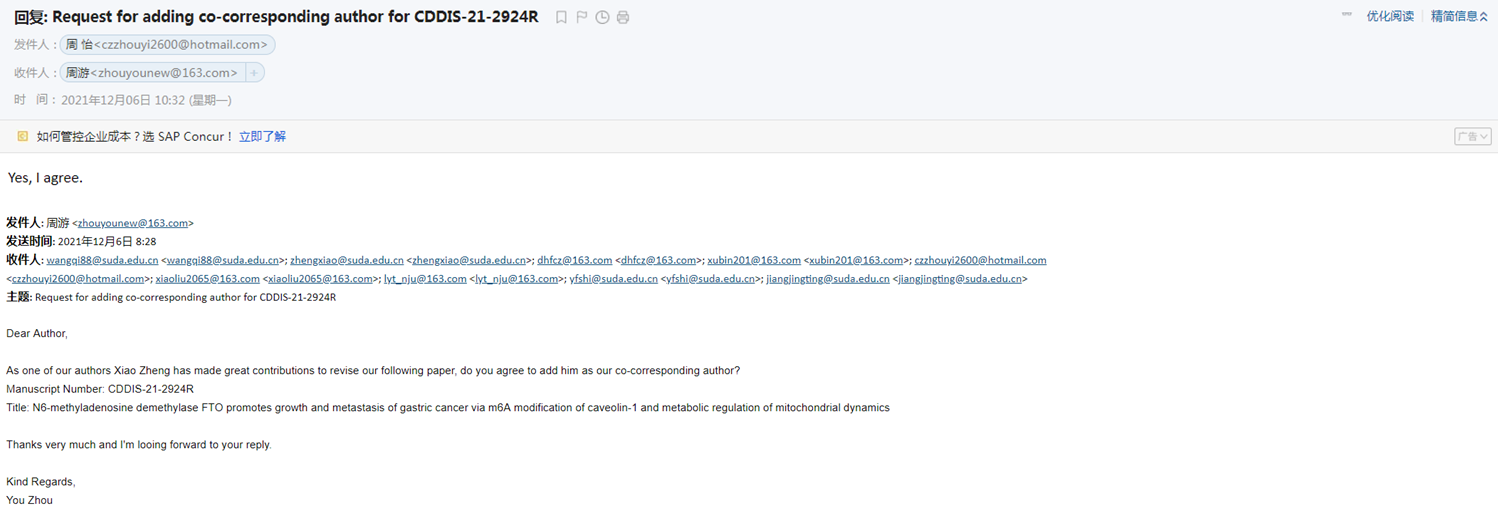


7. Agreement from co-author Jian Liu:


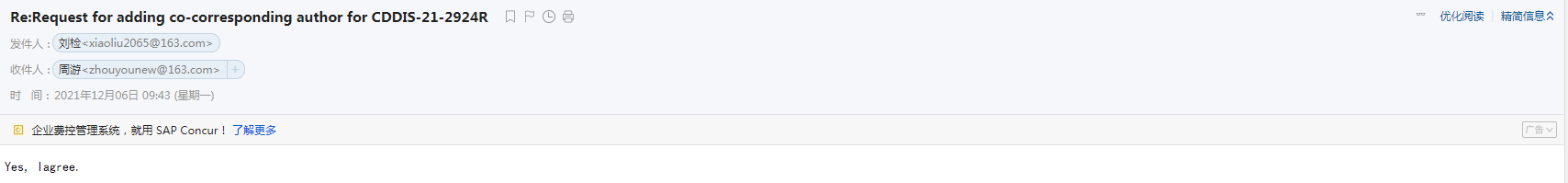

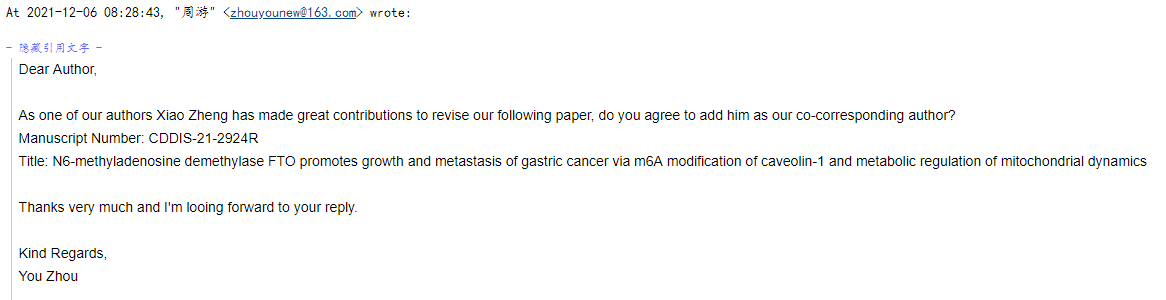


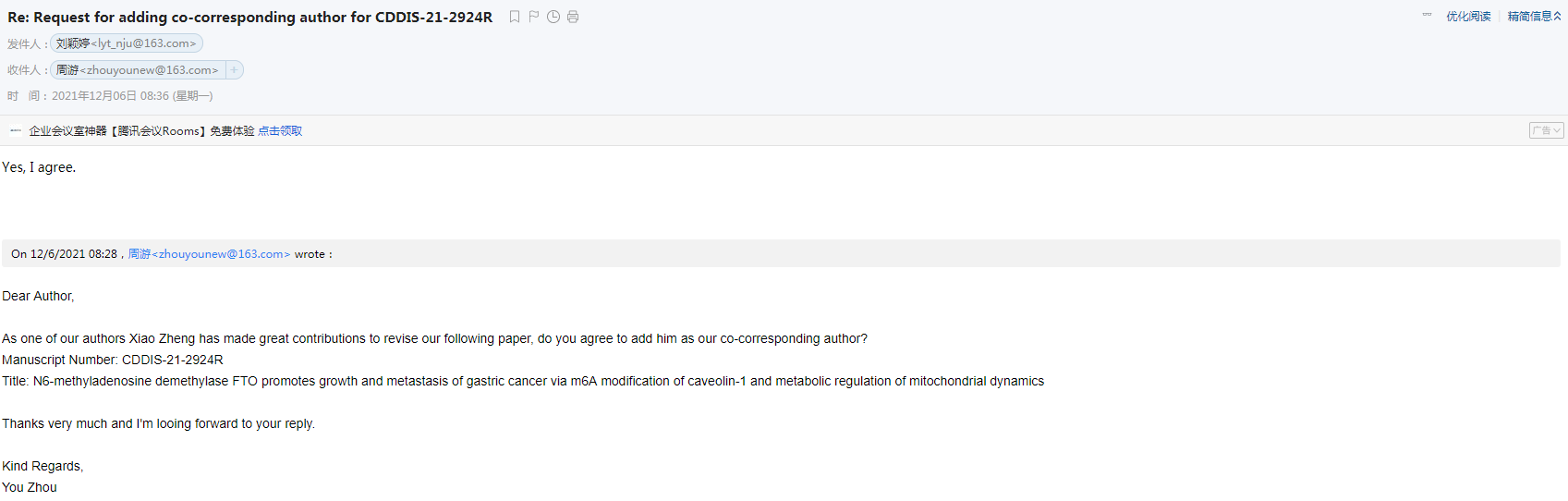
8. Agreement from co-author Yingting Liu:

9. Agreement from co-author Yufang Shi:


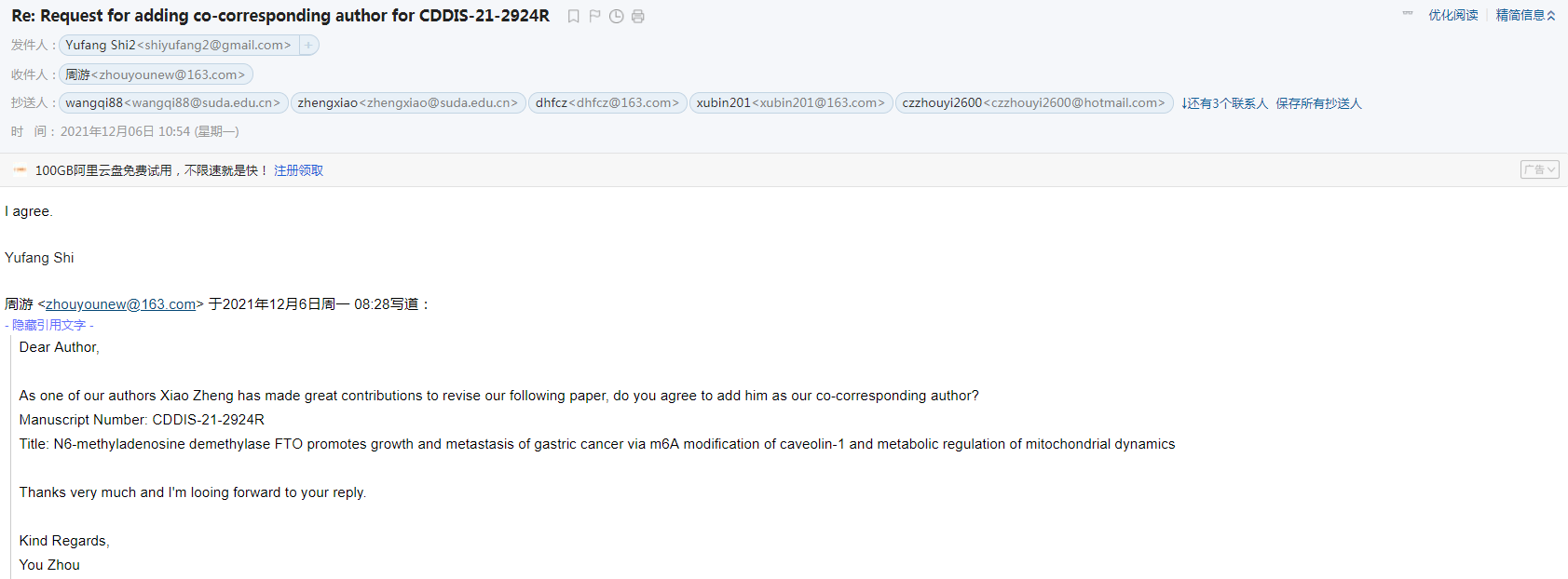


10. Agreement from co-author Jingting Jiang:


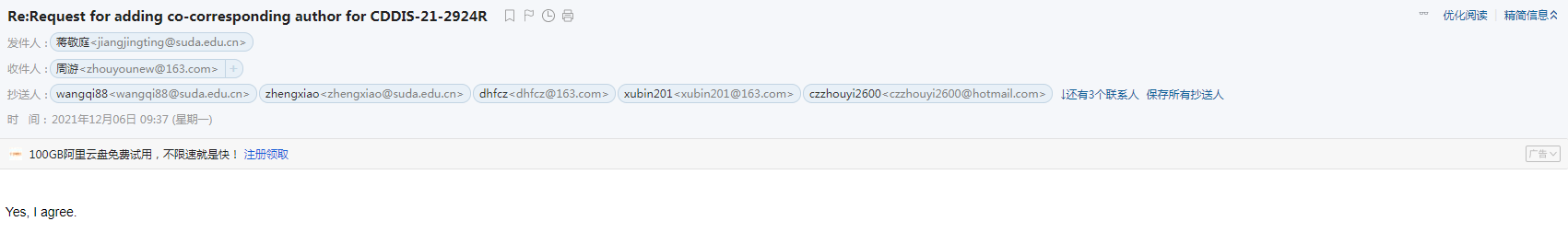

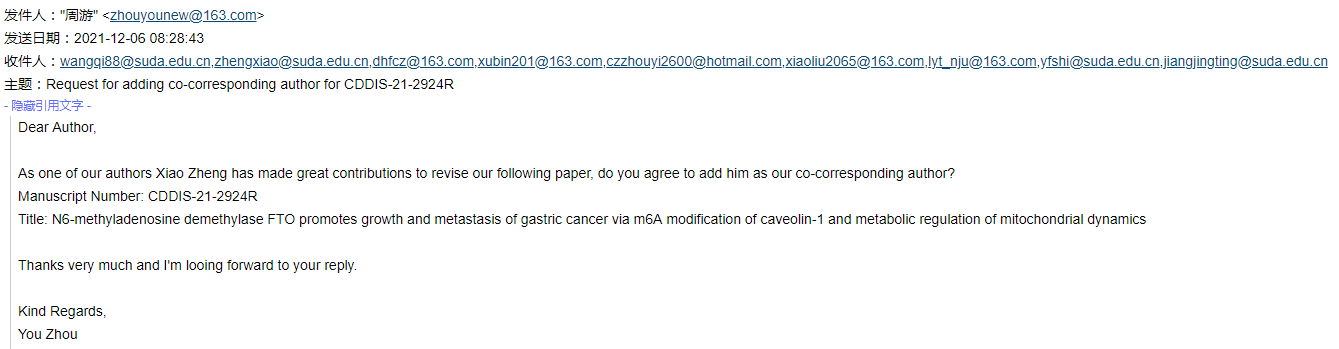

Supplement: Supplementary file 3 — Agreement from co-authors [file 41419_2022_4503_MOESM3_ESM.doc]
