# Supplementary material for: N6-methyladenosine demethylase FTO promotes growth and metastasis of gastric cancer via m6A modification of caveolin-1 and metabolic regulation of mitochondrial dynamics
Source: Cell Death Dis. 2022 Jan 21;13(1):72. doi: 10.1038/s41419-022-04503-7 (PMC8782929; doi:10.1038/s41419-022-04503-7)
Supplement: Supplementary file 4 — Author Contribution Statement [file 41419_2022_4503_MOESM4_ESM.doc]

**AUTHOR CONTRIBUTIONS**

Y.Z., Y.S. and J.J. conceived the study. Q.W., X.Z., B.X. and H.D. performed the experiments. J.L. and Y.L. analyzed and interpreted the data. All authors contributed to biological analysis, interpretation of the results, read, and approved the final version of the manuscript.
