# Supplementary material for: N6-methyladenosine demethylase FTO promotes growth and metastasis of gastric cancer via m6A modification of caveolin-1 and metabolic regulation of mitochondrial dynamics
Source: Cell Death Dis. 2022 Jan 21;13(1):72. doi: 10.1038/s41419-022-04503-7 (PMC8782929; doi:10.1038/s41419-022-04503-7)
Supplement: Supplementary file 6 — Supplementary figure legend [file 41419_2022_4503_MOESM6_ESM.doc]

**Supplementary Figure legends**

**Supplementary Fig. 1** Mitochondrial fission inhibition with Mdivi-1 significantly suppressed the cell proliferation of FTO over-expressed AGS (left panel) and SGC-7901 cells (right panel). N=3, **P* < 0.05 compared with the indicated group.
